# Supplementary material for: Domain engineering of the metastable domains in the 4f-uniaxial-ferromagnet CeRu2Ga2B
Source: Sci Rep. 2017 Apr 10;7:46296. doi: 10.1038/srep46296 (PMC5385885; doi:10.1038/srep46296)
Supplement: Supplementary Information [file srep46296-s1.pdf]

Domain engineering of the metastable domains in the 4f-uniaxial-ferromagnet $CeRu_2Ga_2B$ – Supplementary Material

D. Wulferding,[1,2] H. Kim,[1,2] I. Yang,[1,2] J. Jeong,[1,2] K. Barros,[3] Y. Kato,[3,4] I. Martin,[3,5] O. E. Ayala-Valenzuela,[1,2] M. Lee,[1,6] H. C. Choi,[1,6] F. Ronning,[7] L. Civale,[7] R. E. Baumbach,[8] E. D. Bauer,[7] J. D. Thompson,[7] R. Movshovich,[7] and J. Kim[1,2]

[1]*Center for Artificial Low Dimensional Electronic Systems, Institute for Basic Science, 77 Cheongam-Ro, Nam-Gu, Pohang 37673, Korea*

[2]*Department of Physics, POSTECH, 77 Cheongam-Ro, Nam-Gu, Pohang 37673, Korea*

[3]*Theoretical Division and CNLS, Los Alamos National Laboratory, Los Alamos, NM 87545, USA*

[4]*RIKEN Center for Emergent Matter Science (CEMS), Wako, Saitama 351-0198, Japan*

[5]*Materials Science Division, Argonne National Laboratory, Argonne, IL 60439, USA*

[6]*Department of Chemistry, POSTECH, 77 Cheongam-Ro, Nam-Gu, Pohang 37673, Korea*

[7]*MPA-CMMS, Los Alamos National Laboratory, Los Alamos, NM 87545, USA*

[8]*National High Magnetic Field Laboratory, Tallahassee, FL 32310, USA*

(Dated: December 5, 2016)

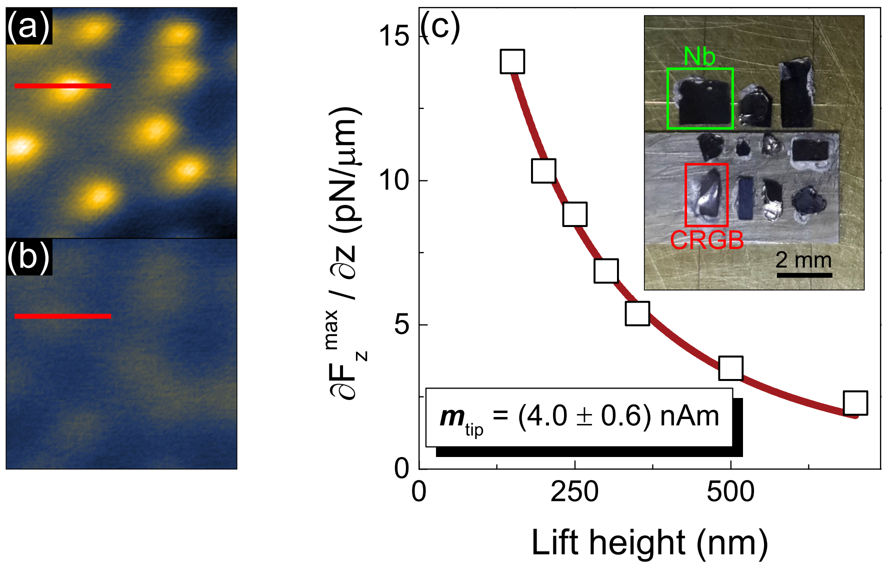

FIG. 1. Supplement Figure 1: Estimation of the tip magnetic moment per unit length. **a,b**, MFM images of Abrikosov vortices in a Nb film obtained at $T = 4$ K at a tip-sample distance of 150 nm and 700 nm, respectively. **c**, Force magnitude as a function of tip-sample distance (open squares). The red solid line is a fit (see text for details). Inset: multi-sample stage, containing various samples investigated during a single cool-down using the same MFM tip.

I. ESTIMATING THE MANIPULATION FORCE

In order to probe the manipulation force of magnetic bubble domains in $CeRu_2Ga_2B$, we first estimate the tip magnetic moment per unit length. We image Abrikosov vortices in a superconducting Nb film at increasing tip-sample distances, which results in a decrease in MFM contrast (see Figs. S1a,b). Each vortex corresponds to one magnetic flux quantum of $\Phi_0 = h/2e$. Next, we plot the force magnitude $\partial F_z^{max}/\partial z$, extracted from line scans through a single vortex, as a function of the tip-sample distance (see Fig. S1c). In a simple monopole-monopole picture, the interaction between the tip magnetic moment and the point-like vortex can be approximated by[1]

$$\frac{\partial F_z^{max}}{\partial z} = \frac{m_{\rm tip} \cdot \Phi_0}{\pi} \times \frac{1}{(d_{\rm ts} + \lambda)^3} \qquad (1)$$

where λ is the London penetration depth ($\approx$ 110 nm at $T = 4$ K), $d_{\rm ts}$ is the tip-sample-distance, and $m_{\rm tip}$ is the tip magnetic moment per unit length. By fitting this approximation to our force gradient data, we obtain $m_{\rm tip} = (4.0 \pm 0.6)$ nAm (see Fig. S1c).

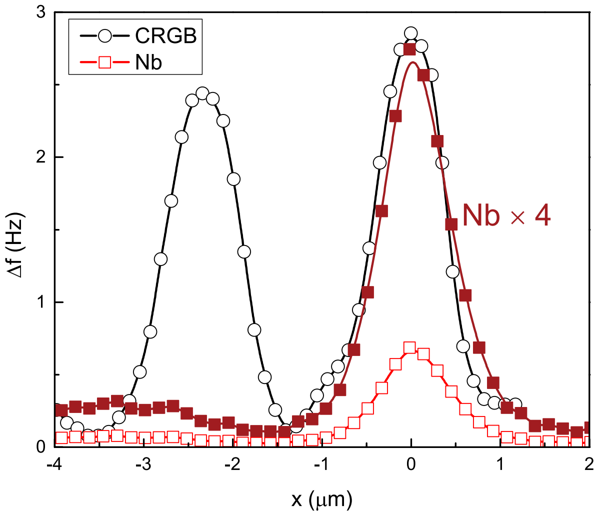

FIG. 2. Supplement Figure 2: Estimation of the magnetic flux through a single bubble domain. Black curve: line profile through a bubble domain in CRGB imaged at a tip sample distance of 300 nm. Bright red curve: line profile through a magnetic quantum flux imaged in Nb at a tip-sample distance of $(d_{ts} + \lambda) = 300$ nm, dark red curve: Nb vortex profile scaled by a factor of 4.

Next, we estimate the magnetic flux through a single magnetic bubble domain in CRGB by comparing it to a well-defined magnetic flux quantum imaged under the same conditions in superconducting Nb, see Fig. S2. Note that our multi-sample stage[2] (see inset in Fig. S1c) allows us to investigate both samples, CRGB and the Nb film, during a single cool-down and with the same MFM tip. This feature enables us to perform comparative studies of various samples using a constant, unchanged tip condition. We find that the bubble diameter in CRGB is comparable to the diameter of individual, isolated vortices in Nb. By scaling the intensity of the flux quantum to coincide with the bubble cross-section, we find that a bubble carries a flux $\Phi_{\rm bubble}$ of roughly 4 times Φ_0. Using these approximations we can now determine the force between tip and bubble necessary for a manipulation process, as described in the main text.

II. MODELING DOMAIN STRUCTURES IN $\mathrm{CERU_2GA_2B}$

To qualitatively model the appearance of bubbles and their evolution, we use an effective energy functional based on a lowest order Ginzburg-Landau expansion,

$$E[\mathbf{S}] = \int \mathrm{d}^3\mathbf{x} \left(\frac{J}{2}\mathbf{S}\cdot\Gamma\mathbf{S} - HS_z - \frac{1}{2}KS_z^2 \right) , \tag{2}$$

where $\mathbf{S}(\mathbf{x})$ is a smooth vector field satisfying $|\mathbf{S}| = 1$ (everywhere except bubble cores). We fix energy units by taking the interaction strength to be $J = 1$. An external field H points in the $\hat{z}$ direction, and K controls the easy-axis anisotropy in the component S_z.

The operator Γ is most easily understood in Fourier space where the interaction term becomes

$$E_{\mathrm{int}} = \frac{1}{2(2\pi)^{-3}} \int \mathrm{d}^3\mathbf{k}\Gamma(\mathbf{k})|\mathbf{S}(\mathbf{k})|^2 ,$$

with $\mathbf{S}(\mathbf{k}) = \int \mathrm{d}^3\mathbf{x}\exp(i\mathbf{k}\cdot\mathbf{x})\mathbf{S}(\mathbf{x})$. In the ferromagnetic Heisenberg model, the choice $\Gamma_{FM} = -\nabla^2$ is standard. The representation in Fourier space, $\Gamma_{FM}(\mathbf{k}) = |k|^2$, has a minimum at $|k| = 0$ and thus favors ferromagnetic configurations. Our aim is to model bubbles of some finite size; for that purpose we introduce higher order derivatives to Γ. The prototypical Swift-Hohenberg model of pattern formation uses $\Gamma_{SH} = (q_0^2 + \nabla^2)^2$ [3], or in Fourier space $\Gamma_{SH}(\mathbf{k}) = (q_0^2 - |k|^2)^2$. The minimum of Γ_{SH} appears at $|k| = q_0$ instead of 0, which introduces a natural modulation length scale, $\lambda = 2\pi q_0^{-1}$. To model the bubble domains seen in CRGB, we select $\lambda \approx 0.5$ μm.

The model above is valid at large length scales, and assumes a smooth field $\mathbf{S}(\mathbf{x})$. To represent bubbles as finite energy defects, our model has to be regularized in the small wavelength, ultra-violet (UV) limit. The core of a bubble singularity may be represented as $\mathbf{S} = \mathbf{r}/|r|$. We can estimate E_{core} for a single bubble using dimensional analysis. The large k scaling of the Swift-Hohenberg operator, $\Gamma_{SH} \sim k^4$, causes the bubble core energy to diverge like the UV cutoff frequency, Λ. The physical lattice constant a (order of 1 nanometer) is not a good regulator, because selecting $\Lambda \sim 1/a$ would lead to a bubble core energy on the order of $1/a$, which is very large compared to the characteristic non-singular part of the configuration energy, $\sim q_0$. In any case, the UV divergence is not physical since there is no reason to believe that $\Gamma(\mathbf{k}) \sim k^4$ at large $|k|$. We regularize the model at large k by choosing

$$\Gamma(\mathbf{k}) = c(|k|)(q_0^2 - |k|^2)^2 \tag{3}$$

$$c(k) = \frac{1}{1 + k^4/\Lambda^4} \tag{4}$$

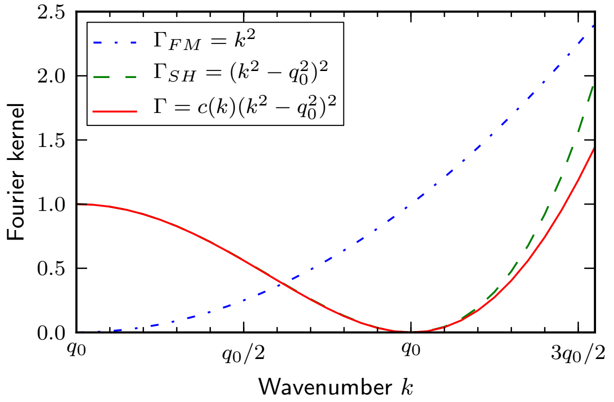

FIG. 3. Supplement Figure 3: Possible Fourier kernels for the interaction term. The choice $\Gamma_{FM} = k^2$ corresponds to the Heisenberg ferromagnet. The choice $\Gamma_{SH} = (q_0^2 - k^2)^2$ corresponds to the Swift-Hohenberg model, and induces modulations on the scale $\lambda \sim 1/q_0$. We use Γ_{SH} with a damping factor $c(k) = (1 + k^4/(2q_0)^4)^{-1}$ to eliminate UV divergences while preserving the physics at $k \sim q_0$.

With this choice, $\Gamma \sim 1$ at frequencies $k \gg \Lambda$. The frequency cutoff Λ controls the bubble core energy. We choose $\Lambda = 2q_0$, where q_0 is the modulation frequency. For this choice, the bubble energy is comparable to the energy of the entire bubble configuration. As shown in Fig. S3, our choice of $\Lambda = 2q_0$ is sufficiently large that Γ is nearly unaffected at its minimum at $k \sim q_0$.

To summarize our theoretical model, it is a minimal one that contains just the four necessary physical parameters: (1) external field H, (2) anisotropy K, (3) modulation frequency q_0, and (4) finite bubble core energy via the frequency cutoff Λ. An alternative regularization of the bubble core energy is to "soften" the spins by lifting the restriction $|\mathbf{S}| = 1$.

The form of the interaction that we constructed is essentially phenomenological, and motivated by the CRGB data. However, we illustrate one scenario of how such an interaction may come about. Consider that the interaction between Ce moments in CRGB is due to two primary interactions: nearest neighbor antiferromagnetic superexchange[4] and longer-range oscillatory in space RKKY interaction[5–7]. Let us show that combining these two interactions one can naturally obtain a long-wavelength modulation. In momentum space,

$$J_{AF}(k) = J_0[\cos(k_x a) + \cos(k_y a) + \cos(k_z a)] \quad (5)$$

$$J_{RKKY}(k) = -J_1 \chi(k), \quad (6)$$

where $\chi(q)$ is the Lindhard function[8]. Expanding around $k = 0$ and dropping an irrelevant constant term, we obtain

$$J_{tot} = A\left[(k/2k_F)^2 + (k/2k_F)^4/5 + \ldots\right] + B\left[-(ka)^2 + (k_x^4 + k_y^4 + k_z^4)/12 + \ldots\right].$$

By tuning parameters A and B, we can cancel the k^2 terms leaving $J \propto k^4/(2k_F)^2/5 + (k_x^4 + k_y^4 + k_z^4)a^2/12$. Allowing the quadratic AF term to slightly dominate, and taking $k_F a \ll 1$, we obtain the desired nearly isotropic form of the interaction peaked at a finite but small value of k.

To perform simulations on this model, we evolve the field $\mathbf{S}(\mathbf{x}, t)$ according to the Langevin equation

$$\frac{\partial \mathbf{S}}{\partial t} = -\frac{\delta E}{\delta \mathbf{S}} + \sqrt{2k_B T}\eta, \tag{7}$$

where $k_B T$ is the temperature in scaled energy units and $\eta(\mathbf{x}, t)$ is Gaussian white noise with moments $\langle\eta(\mathbf{x}, t)\rangle = 0$ and $\langle\eta(\mathbf{x}, t)\eta(\mathbf{x}', t')\rangle = \delta(\mathbf{x} - \mathbf{x}')\delta(t - t')$. The functional derivative of energy is

$$\frac{\delta E}{\delta \mathbf{S}} = \int \mathrm{d}^3\mathbf{x}\Gamma\mathbf{S}(\mathbf{x}) - H\hat{z} - KS_z\hat{z}. \tag{8}$$

To satisfy the constraint $|\mathbf{S}| = 1$, we modify the time evolution with a Lagrange multiplier term $\partial\mathbf{S}/\partial t \to \partial\mathbf{S}/\partial t + \lambda\mathbf{S}$, where λ is to be solved self-consistently. Assuming ergodicity, this Langevin equation generates fields $\mathbf{S}$ with the appropriate Boltzmann distribution, $P[\mathbf{S}] \propto \exp(-\beta E)$. In the spirit of time-dependent Ginzburg-Landau modeling, we will interpret the evolution of $\mathbf{S}$ as a qualitative description of the *non-equilibrium* dynamical evolution of $\mathbf{S}$.

We integrate the Langevin equation using an implicit numerical scheme for stability, and alternating between Fourier and real space for efficiency. The steps are:

1. Use the Fast Fourier Transform (FFT) to calculate $\mathbf{S}(\mathbf{k}, t)$. In Fourier space, construct $\mathbf{T}(\mathbf{k}, t) \equiv (1 + \Delta t\Gamma(\mathbf{k}))\mathbf{S}(\mathbf{k}, t)$, using the functional form for $\Gamma(\mathbf{k})$ given in Eq. (3).

2. Use a reverse FFT to calculate $\mathbf{T}(\mathbf{x}, t)$.

3. Update $\mathbf{S}(x, t)$ according to

$$\mathbf{S}(\mathbf{x}, t + \Delta t) = \mathbf{T}(\mathbf{x}, t) + \Delta t\hat{z}(H + KS_z) + \sqrt{2k_B T\Delta t}\xi_{\mathbf{x},t}$$

where each $\xi_{\mathbf{x},t}$ is a Gaussian random number with unit variance.

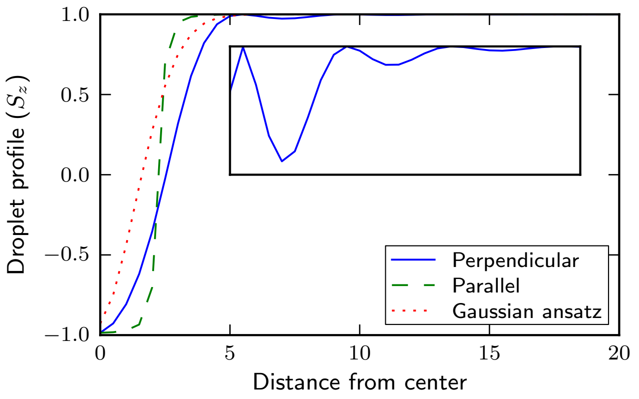

FIG. 4. Supplement Figure 4: The profile of S_z as a function of distance from the bubble center. The profile decays quickly in both parallel and perpendicular directions, consistent with the numerically observed weak bubble-bubble interaction. The parallel direction, passing through the bubble defect, has a sharp jump in S_z at $r \approx 2$ at the singularity. A limitation of our Gaussian ansatz is that it is radially symmetric, and does not contain a sharp jump in S_z. Inset: Zoom-in around $S_z = +1.0$.

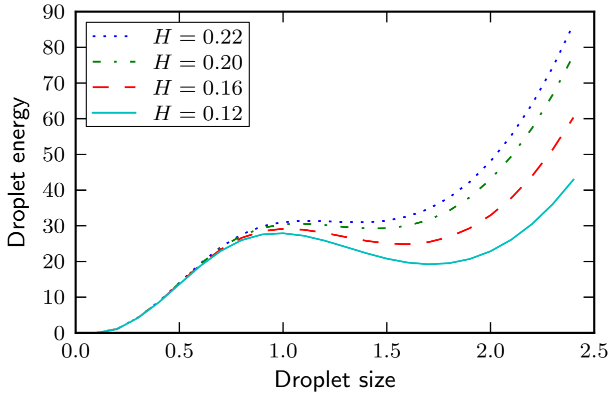

FIG. 5. Supplement Figure 5: Energy of a single bubble as a function of linear size, using the Gaussian ansatz. A well defined energetic minimum indicates preferred bubble size. At fields $H \gtrsim 0.22$ the bubble is unstable to decreasing size σ and dynamically implodes. At fields $H \lesssim 0.12$ the bubble is unstable to deconfinement.

4. Normalize $\mathbf{S}(\mathbf{x}, t + \Delta t)$ to satisfy the constraint $|\mathbf{S}| = 1$.

The bubbles we observe are locally stable, but do not globally minimize the energy. For these system parameters, the true energy minimum is actually the ferromagnetic state

$\mathbf{S} \approx +\hat{z}$. To understand the energetics of a single bubble, we apply the ansatz,

$$S_z = 1 - 2e^{-r^2/2\sigma^2}$$
$$S_x = \cos\phi\sqrt{1 - S_z^2}$$
$$S_y = \sin\phi\sqrt{1 - S_z^2}$$

where r is the radial distance from the origin and ϕ is the azimuthal angle. Our Gaussian ansatz has a single parameter σ, the linear bubble size. For this ansatz, $S_z = 0$ at radial distance $r_0 = \sigma\sqrt{2\ln 2} \approx 1.18\sigma$. Our ansatz does not capture the bubble defects precisely. Instead, the entire z axis is singular because S_x and S_y are ill-defined on this line. In an actual bubble of minimal energy, we would observe $S_z = -1$ along the entire z axis when $r < r_0$, and $S_z = +1$ when $r > r_0$. This discrepancy is illustrated in Fig. S4, where a real bubble of minimum energy is compared to the Gaussian ansatz.

The Gaussian ansatz correctly predicts bubble annihilation at large external field H. In Fig. S5 we plot the ansatz energy as a function of bubble size σ at fixed $K = 0.25$ and varying H. The pronounced energetic minimum indicates a preferred bubble size that decreases slightly with increasing field H. At fields $H \gtrsim 0.22$ the local minimum is removed and the bubble annihilates, leaving behind pure ferromagnetic alignment. This behavior is consistent with what we observe in Langevin dynamics simulations, Eq. (7). Our Langevin simulations also demonstrate that the bubble is unstable to deconfinement (and extension of the string) when $H \lesssim 0.12$.

---

[1] Auslaender, O. M. *et al.* Mechanics of individual isolated vortices in a cuprate superconductor. *Nature Phys.* **5**, 35 (2009).

[2] Yang, J. *et al.* Construction of a ^{3}He magnetic force microscope with a vector magnet. *Rev. Sci. Instrum.* **87**, 023704 (2016).

[3] Swift, J. & Hohenberg, P. C. Hydrodynamic fluctuations at the convective instability. *Phys. Rev. A* **15**, 319328 (1977).

[4] Anderson, P. W. Antiferromagnetism. theory of superexchange interaction. *Phys. Rev. 79*, 350356 (1950).

[5] Ruderman, M. A. & Kittel, C. Indirect exchange coupling of nuclear magnetic moments by conduction electrons. *Phys. Rev.* **96**, 99102 (1954).

[6] Kasuya, T. A theory of metallic ferro- and antiferromagnetism on Zener's model. *Prog. Theor. Phys.* **16**, 4557 (1956).

[7] Yosida, K. Magnetic properties of Cu-Mn alloys. *Phys. Rev.* **106**, 893898 (1957).

[8] Lindhard, J. On the properties of a gas of charged particles. *Kgl. Danske Videnskab. Selskab Mat.-fys. Medd.* **28** (1954).
